# Supplementary material for: The impact of bite force on the stability of dental implants
Source: PLoS One. 2026 Jan 20;21(1):e0340355. doi: 10.1371/journal.pone.0340355 (PMC12818635; doi:10.1371/journal.pone.0340355)
Supplement: S2 File — doi: https://doi.org/10.6084/m9.figshare.29364278.v1. (DOCX) [file pone.0340355.s002.docx]

| STROBE Statement—checklist of items that should be included in reports of observational studies | | | | |
| --- | --- | --- | --- | --- |
|  | Item No. | Recommendation | Page  No. | Relevant text from manuscript |
| **Title and abstract** | 1 | (*a*) Indicate the study’s design with a commonly used term in the title or the abstract | 1 | Title: The impact of bite force on the stability of dental implants |
|  |  | (*b*) Provide in the abstract an informative and balanced summary of what was done and what was found | 2 | ABSTRACT  Background: Since dental implants (DI) are endo-osseous implants that are inserted into the bone and periodontium, which ultimately support the occlusal load, a patient's bite force (BF) may overburden the supporting DI, leading to bone loss and DI failure. This study purpose to explore the effect of the BF on DIs of different genders, to investigate DI stability in both gender, to assess the correlation between BF and DI stability, and to determine the impact of the BF on stability and longevity of DIs.  Methods: The current cohort study involved 80 individuals of both genders who had lost some teeth and needed DIs for the anterior and posterior regions of the jaws. After the insertion of the DIs, their BF was examined by Loadstar™ sensors and the DI stability was monitored at three visits by these individuals.  Results: The data on the anterior and posterior occlusal forces (OFs) for the male and female participants after the insertion of the DIs were analysed. On average, the females exhibited a lower anterior OF compared to the males. Similarly, the average posterior OF in the females was lower than in the males at the respective three visits. Adding the average anterior OF in the females and males was lower than posterior OF in the females and males. Meanwhile, the present study found an increase in the stability of the anterior DIs of the female participants compared to the males, and the stability of the posterior DIs of the females was greater than the males at the respective three visits. The stability of the anterior DIs of the females and males was greater than posterior DIs of the females and males. The correlation between the OF and DI stability was significantly inverse in the male (p:0.002) and female(p:0.012) participants.  Conclusions: The lower BF in the anterior regions of females and males could play a potential role in the greater stability and longevity of DIs. The success of DIs that are loaded early is dependent on the OF. The BF may be crucial in determining the best DI therapy.  Keywords: Bite Force, Dental Implant, Implant Stability, Loadstar Sensors. |
| Introduction | | | |  |
| Background/rationale | 2 | Explain the scientific background and rationale for the investigation being reported | 3-4 | Dental implants (DIs) have become a popular and effective option for replacing missing teeth [1,2.] One of the most important factors that affect the success of DIs is the occlusal force (OF) or the force that is applied to the DI by opposing teeth during biting and chewing [3,4]. The occlusal force (OF) can have both positive and negative effects on the DI, depending on the amount and direction of the force [5,6].Early loading of a DI, which involves placing the DI rapidly after surgery, is significantly popular in the field of DI dentistry. However, the impact of the OF on an early-loaded DI is still a topic of debate and research [7]. Several studies indicate that initiating early loading with a carefully regulated OF can improve the process of osseointegration and result in favourable results for the DI [8-10]. Nevertheless, an excessive or unregulated OF can lead to the failure of the DI, bone resorption, and fracture of the DI. The impact of the OF on an early-loaded DI can be influenced by several factors, including the DI design, DI site, bone quality, and the nature and intensity of the OF [11]. Dental implants (DIs) inserted into soft bones are more prone to injury due to the OF compared to those inserted into thick bones [12,13]. Applying a regulated amount of pressure on the teeth can improve the integration of the DI with the surrounding bone and increase the chances of a good outcome. However, an excessive or unregulated force might cause the DI to fail and lead to bone loss [14].Bite force (BF) is the pressure applied by the jaws while chewing and biting. It is impacted by variables such as the quantity and arrangement of the teeth, the force exerted by the muscles involved, and the general oral well-being of an individual. An optimal BF is essential for effective chewing and ensuring the durability of dental restorations, such as DIs. It differs from person to person and greatly impacts the DI system [15,16].The DI abutment transmits the BF to the DI, which in turn transfers it to the crown denture or other prosthetic parts. It is crucial to assess and take into consideration the BF when planning and creating DI-supported restorations [17].  The BF is determined by several factors, including the characteristics relating to the patient and the prosthesis. The factors that are dependent on the patient are the patient’s age, gender, occlusal habits, parafunctional behaviours, such as teeth grinding and overall health of the stomatognathic system [18-20], while the prosthesis-related factors include the design, material, occlusal arrangement, and number of teeth supported by the DI [21].A restored DI transmits multidirectional forces that alter the amount of axial, nonaxial, and transversal loads throughout the chewing process and movements of the jaw, affecting the connection between the DI and the bone [22].Modifications to the design of the neck of the DI, which gives rise to bone reabsorption, often take place in this area of highly concentrated mechanical forces [23]. The changes made to the neck of the DI are intended to lessen pressures like the forces of tension and shear in the cortical area [24,25].The success and long-term were usefulness of DIs [26,27]. While DIs offer numerous benefits, such as improved aesthetics and functionality, the impact of the BF on their long-term success undetected. The research question is the BF impact on stability and longevity of DIs. This study aims to explore the effect of the BF on DIs of different genders, to investigate DI stability in both genders, and to assess the correlation between BF and DI stability. The null hypothesis is that the BF could not a significantly impact on stability and longevity of DIs. |
| Objectives | 3 | State specific objectives, including any prespecified hypotheses | 4 | This study aims to explore the effect of the BF on DIs of different genders, to investigate DI stability in both genders, to assess the correlation between BF and DI stability, and to determine the impact of the BF on stability and longevity of DIs. |
| Methods | | | |  |
| Study design | 4 | Present key elements of study design early in the paper | 4 | Study design, this cohort study used a prospective approach to analyse the clinical information and records of a group of patients who needed DI placements between 20 February 2021 to 20 June 2024. All procedures performed in this study involving human participants were in accordance with the Declaration of Helsinki and its later amendments for human research. The study was conducted in accordance with and approved by the Ethics Committee of College of Dentistry, University of Al-Ameed, Iraq (#52017). Written informed consent was obtained from all subjects and/or their legal guardian (s), where, all adult participants entered the study after they were received full information about the nature, aims and processes of the study before signing an informed written Consent form. |
| Setting | 5 | Describe the setting, locations, and relevant dates, including periods of recruitment, exposure, follow-up, and data collection | 4 | The study included 80 patients at the Maxillofacial Surgery Department of the Dentistry College at the University of Al-Ameed who needed DIs during the study period with three follow up visits. First visit during the day of insertion the crown on abutment of implant (immediately loading),t, second visit at six months following implant insertion, and third visit after one half year following insertion. |
| Participants | 6 | (*a*) *Cohort study*—Give the eligibility criteria, and the sources and methods of selection of participants. Describe methods of follow-up  *Case-control study*—Give the eligibility criteria, and the sources and methods of case ascertainment and control selection. Give the rationale for the choice of cases and controls  *Cross-sectional study*—Give the eligibility criteria, and the sources and methods of selection of participants | 5 | The inclusion criteria were male and female patients aged 25-40 years, who had sufficient bone volume for DI placements and had natural occlusions opposing to implants that need inserting, were understand and sign an informed consent form and were followed up postoperatively visits. Patients with a history of systemic diseases affecting bone metabolism, periodontitis, cigarette smoker and drinking alcohol were excluded from the study. Moreover, the exclusion criteria included; they had missing permanent teeth, implant and restored with crown of opposing occlusion. |
|  |  | (*b*) *Cohort study*—For matched studies, give matching criteria and number of exposed and unexposed  *Case-control study*—For matched studies, give matching criteria and the number of controls per case | 5 | The study included 80 patients of both genders who had lost some teeth and needed DIs for the anterior and posterior regions of the jaws. |
| Variables | 7 | Clearly define all outcomes, exposures, predictors, potential confounders, and effect modifiers. Give diagnostic criteria, if applicable | 6 | Outcome Measures  The main objective of the study was to determine the relationship between the BF and the success of DIs. The success of a dental implant (DI) is described as the maintain DI stability and lack of any failure of the DI due to loss of bone around the DI throughout the time that followed. The secondary outcomes included changes with gender and the DI location in the oral cavity that affected the BF on the DI and stability.  The demographic information collected from the participants included: (a) Age (range, 25‐40 years); ; and (c) gender (both man and woman). |
| Data sources/ measurement | 8* | For each variable of interest, give sources of data and details of methods of assessment (measurement). Describe comparability of assessment methods if there is more than one group | 5-6 | Data Collection  Relevant clinical information, such as the patient’s characteristics after placement with a DI by specialist, medical background, X-ray pictures, and scans of the inside of the mouth, were obtained from the computerised medical records and documents of the patient. A Loadstar™ sensor was used to collect the BF values of posterior and anterior implants areas [28].. This sensor offers various force-measuring solutions with updated data rates of up to 50 KHz, making it suitable for applications that require the BF to be noted soon after the operation and during consultation intervals. Regular monitoring of the BF and DI stability was crucial in the post-implantation phase. Loadstar™ sensor records were utilised to assess the OF and identify any abnormalities or imbalances, while Osstell Implant Stability Quotient (ISQ) technology was used to evaluate the DI stability by examining the resonance frequency of the DI. The patients were also educated on proper oral hygiene practices, including avoiding excessive BF on the DI, maintaining regular dental visits, and promptly addressing any signs of discomfort or changes in the bite.  Dental Implant (DI) Characteristics  Information on the DIs of the research population, which were specifically manufactured by Strumman and Medintika, (Straumann® Dental Implant System.USA, Medintika® Dental Implant.Germany) was carefully recorded, including their size (4.0*10, 3.5*11, 3.3*10, or 3.8*9), surface features, and prosthetic elements. Any alterations to the shape of the DI by modifications to the design of the neck of the DI or the process of surgery for improving the spread of the load and enhancing the stability were observed.  Bite Force (BF) Assessment  Bite force (BF) assessments were acquired utilising a Loadstar™ sensor(Loadstar sensor, DI-100U,16-bit load cell interface, Fremont. California) (Figure 1), which offers various force measurement devices with updated data rates of up to 50 KHz. This capability might be advantageous for applications such as failure strength testing or material characterisation. The BF values were tested within the range of 100-400 N at particular time intervals. This systematic method was observed to guarantee uniformity and dependability in the assessment of the BF.  Stability measurements  The method for monitoring implant stability during healing and immediately loading is based on resonance frequency analysis (RFA) applied to the implant–bone interface. Devices using this method contain a transducer peg, which is connected to the implant and excited by magnetic waves over a range of frequencies. The frequency of the resultant vibration is automatically translated into an index called the implant stability quotient (ISQ), with values ranging between 0 and 100. The RFA values are a measure of the deflection of the implant–bone complex by the lateral forces applied by the transducer and reflect the multidirectional fixation strength. The stability of each implant (one measurement from each of the 3 different directions) was measured with the Osstell (Integration Diagnostics AB, Göteborg, Sweden). The Osstell system is a magnetic detection device. RFA devices after the transducer (smartpeg) was screwed to the implant to obtain the ISQ. |
| Bias | 9 | Describe any efforts to address potential sources of bias |  | Although it is likely that some suitable individuals were overlooked since the patients were not selected randomly because the study design method, this was minimized by enrolling all patients using a sequential strategy. |
| Study size | 10 | Explain how the study size was arrived at | 4 | Sample Size Power Analysis  The sample size, calculated by G power, comprised 18 individuals in each group at a power of 80 and α probability of 0.05. The total number of participants was around 80 to avoid patient dropouts, and these were divided into four groups. |
| Quantitative variables | 11 | Explain how quantitative variables were handled in the analyses. If applicable, describe which groupings were chosen and why | 6 | Determining bite forces of patients and stability measurements of implants. The assessing of differences in bite force and DI stability in males and females at anterior and posterior area of jaws among three followed-up sessions postoperatively. Then investigate the association between the BF and DI stability, |
| Statistical methods | 12 | (*a*) Describe all statistical methods, including those used to control for confounding | 6-7 | Descriptive statistics through GraphPad® Prism 9.5.1 were used to summarise the patient demographics, BF measurements, and DI stability measurements. The continuous variables were expressed as the mean standard deviation (±0.05), while the categorical variables were presented as frequencies and percentages. |
|  |  | (*b*) Describe any methods used to examine subgroups and interactions | 7 | Comparative analyses, such as an analysis of variance (ANOVA), were performed to assess differences in the BF and DI stability in males and females at anterior and posterior area of jaws among three postoperative time points. A Pearson correlation was done to investigate the association between the BF and DI stability, while a linear regression analysis was conducted to identify the changes in the BF associated with the stability of the DI. |
|  |  | (*c*) Explain how missing data were addressed |  | This study doesn’t contain missing data |
|  |  | (*d*) *Cohort study*—If applicable, explain how loss to follow-up was addressed  *Case-control study*—If applicable, explain how matching of cases and controls was addressed  *Cross-sectional study*—If applicable, describe analytical methods taking account of sampling strategy | 7 | If any patient in this study missed a follow-up visit, the author would call the patient to revisit with a new appointment. |
|  |  | (*e*) Describe any sensitivity analyses | 7 | Linear regression analysis was conducted to identify the changes in the BF associated with the stability of the DI. |
| Results | | | | |
| Participants | 13* | (a) Report numbers of individuals at each stage of study—eg numbers potentially eligible, examined for eligibility, confirmed eligible, included in the study, completing follow-up, and analysed | 7 | The current study is enrolled 80 individuals need dental implant for anterior and posterior area in the jaws in both gender. |
|  |  | (b) Give reasons for non-participation at each stage |  | the patients non-participation at this study because they don’t willing |
|  |  | (c) Consider use of a flow diagram |  | non |
| Descriptive data | 14* | (a) Give characteristics of study participants (eg demographic, clinical, social) and information on exposures and potential confounders | 7 | Descriptive data of patient demographics (age and gender), occlusal force measurements, and DI stability. |
|  |  | (b) Indicate number of participants with missing data for each variable of interest |  | non |
|  |  | (c) *Cohort study*—Summarise follow-up time (eg, average and total amount) | 7 | The average of three visits of occlusal forces and stability of implants |
| Outcome data | 15* | *Cohort study*—Report numbers of outcome events or summary measures over time | 7-8 | Table 1 presents the data on the anterior and posterior OF for the male and female participants after receiving the DI. On average, the female participants exhibited a lower anterior OF compared to the males. Similarly, the average posterior OF in the female patients was lower than in the males at the three visits (Figure 2). Adding the average anterior OF in the females and males was lower than posterior OF in the females and males. These findings suggested that the difference in the OF was based on gender. The stability at the anterior and posterior DIs for the male and female participants at the three visits is shown in Table 2 and Figure 3. The present finding showed that the stability of the anterior DI increased for the female participants compared to the males. Likewise, the average stability of the posterior DIs in the female participants was superior to that of the males, as illustrated in the respective three visits. The stability of the anterior DIs of the females and males was greater than posterior DIs of the females and males. The result showed that gender differences may have played a potential role in the stability and longevity of DIs. |
|  |  | *Case-control study—*Report numbers in each exposure category, or summary measures of exposure |  |  |
|  |  | *Cross-sectional study—*Report numbers of outcome events or summary measures |  |  |
| Main results | 16 | (a) Give unadjusted estimates and, if applicable, confounder-adjusted estimates and their precision (eg, 95% confidence interval). Make clear which confounders were adjusted for and why they were included |  | This study was not have confounder for adjusting it, |
| Other analyses | 17 | Report other analyses done—eg analyses of subgroups and interactions, and sensitivity analyses | 8-9 | The data showed that the correlation between the OF and DI stability was significant in the male and female participants (Table 3). The result revealed an inverse correlation between the OF and DI stability (Figure 4). |
| Discussion | | | | |
| Key results | 18 | Summarise key results with reference to study objectives | 9-11 | The bite force (BF) may be a crucial factor on prosthetic design, and stability of DIs, particularly in individuals who can produce extremely high occlusal loads [29]. The current study found that the OF in the anterior DIs of the females and males was lower than in the posterior DIs. Thus, the BF of the patient may be a concern for a good result in the long term, especially in the maxillary anterior DI in both genders, since the facial cortical bone may be most susceptible to overload. The bite force (BF) could be a crucial planning factor. The size, quantity, and occlusal design elements of the DI that can effectively withstand the load may be required for a patient who creates an excessive load [30]. A high BF capacity may indicate a high risk for a late component fracture [31]. The BF on the posterior regions of males and females is always larger than on the anterior regions. Different occlusion circumstances primarily influence the amplitude and direction of the BF. The DI and the entire mandible underwent noticeably increased stress under occlusion [32]. The DI length, location, an anterior-directed occlusal scheme, splinting, and ridge expansion augmentation may be used to improve osseous support or deflect or reduce the OF [33]. Even with poor anatomical bone characteristics, a patient with a moderate BF may be able to have a satisfactory long-term result [34,35].The study revealed that there was an increase in the stability of the anterior DIs of the female and male participants compared to the posterior DIs. Hence, to effectively incorporate the DI into the bone, it is first necessary to establish the primary and secondary stability of the DI. The success rate of dental implants (DIs) may be highly dependent on the stability of the patient's DI [36]. However, early failure can happen in a DI for several reasons, even before the restorative component is inserted. Factors that have been positively associated with a higher risk of failure include advanced age, diabetes, cigarette smoking, and lengthier DIs [37].There are two types of DI stability: primary stability, which involves the mechanical connection of the DI to the bone around its placement, and secondary stability, which involves the tissue reaction to the DI and the subsequent bone remodelling events [38]. The anterior region of the mouth consists of a dense trabecular bone with a thick cortical plate compared to the posterior part of the mouth. Therefore, the primary stability is higher in this region of the mouth [39].The correlation between the OF and DI stability was significant in males and females concerning how the OF initiates bone loss around the DI or how it relates to DI stability. Even after the DI has been joined with the bone, the OF can affect the DI-bone contact and the cells that rebuild the bone in various ways, which can affect whether or not the integration is maintained [40].  The result indicated an inverse correlation between the OF and DI stability. This finding suggested that the location of the posterior DI and a higher BF were the most essential issues governing the effectiveness of the masticatory system and decreasing the stability of the DI [41].Primary stability has also been demonstrated to be influenced by the diameter, surface features and length of implants. The present study dental implants used with size (4.0*10, 3.5*11, 3.3*10, or 3.8*9) were highly primary and secondary stability. More surface area and a stronger mechanical connection to the tissue around it are provided by roughness surfaces of implants [42]. Sandblasted implant surfaces facilitate osteogenesis by increasing osteoblast proliferation and cellular metabolism, according to research conducted in vitro [43,44]. Research has demonstrated the presence of surface response and interaction of cells [45]. Compared to implants with a machining surface, those with acid-etched coatings can achieve a much better bone-to-implant contact in areas with low quality of bone [46]. Experimental evidence has demonstrated in situations when the amount of bone is limited and implants with diameters below 3.0 millimeters offer adequate initial stability [47]. According to Aparicio et al.'s study on RFA procedures, variables such supracrestal implant length, abutment length, and upper or lower jaw bone density appear to affect RFA values [48]. The dimensions of implant outcome influence on stability is consistent with the Raz et al. study's findings, which show that the stability evaluations show greater values for longer implants than for shorter ones, and for densely embedded bone as opposed to softer bone [39]. Implants with a severe thread pattern may improve initial stability [27]. The stabilityvalues of tapering implants were consistently higher than those of cylinder implants [29].These changes involve platform switching and micro threads of DIs used in this study. Compared to non-platform switching designs, platform-switching setups have demonstrated efficient stress performance and reduced the possibility of overloading [49]. The maximum von Mises, compressive, and tensile stresses are reduced when oblique forces are applied to a DI with a platform-switching design compared to a traditional design [50]. The palatal side of the platform and the entire implant surface get a redistribution of the pressures that are moved from the compact bone area to the cancellous bone area [20]. The maximal stresses at the cortical region were lower with platform-switching implants than with conventional implants. Implants with platform switching decreased stress by 40% when subjected to oblique loads and 36% when subjected to axial stresses [21].After osseointegration and throughout the duration of their use, it is acknowledged that all implants exhibit some degree loss of bone. According to several claims, the addition of microthreads or "retention grooves" to the implant's neck may help to distribute stress and lessen the amount of bone loss that occurs after install [51]. In practice, the surgical method and the use of platform switching are linked to the preservation of crestal bone [52]. Additionally, it appears that the progressive thread pattern reduces crestal bone compression process, hence preventing crestal bone loss [52]. |
| Limitations | 19 | Discuss limitations of the study, taking into account sources of potential bias or imprecision. Discuss both direction and magnitude of any potential bias | 12 | The limitation of this study was that patients who smoked and were hypertensive were not included to avoid bias. The small sample size was included in this study that cause non-significant differences between study groups, and the increase of subject numbers that required more time for work and follow up visits over the period of study decision. This study was absence of a control group to compare with it because exposed the inclusion criteria of the cohort subjects was not included. |
| Interpretation | 20 | Give a cautious overall interpretation of results considering objectives, limitations, multiplicity of analyses, results from similar studies, and other relevant evidence | 11-12 | The von Mises stress distribution on the DI system demonstrated that the high stresses on the DI resulted from the action of external forces that primarily occurred close to the DI, where it made contact with the abutment [53]. As a result, when the tooth experienced external stress, the neck of the DI was immediately deformed. Hooke's law predicted that a lot of tension would be produced in this region [54]. The alveolar bone of the DI next to those that were impacted by external forces was also discovered to be subject to significant stress as a result of deformation [55]. Additionally, it was clear from looking at the tension on the abutment and abutment screw that the significant stress on the abutment originated from its intersection with the DI [56,57]. The high stress on the abutment screw developed at the point where the screw head was attached to the abutment and where the geometric shape of the screw head and screw bent [58,59]. Therefore, excessive stress caused by the BF should be avoided in the design of the abutments and abutment screws. Otherwise, the DI system may be worn down since the patient will be chewing with it for a prolonged period. By analysing the BF, dental experts can better understand the functional strain exerted on the DI system [60]. This will facilitate the choice of suitable DI parts and materials that can withstand the stresses generated during chewing and biting [61,62]. Clinicians can avoid premature mechanical failures such screw loosening, abutment fracture, or even DI failure by taking into the patient's BF [63-65]. Understanding the distribution of the BF can also help to achieve occlusal stability, reduce possible issues, and enhance patient comfort [66,67]. |
| Generalisability | 21 | Discuss the generalisability (external validity) of the study results | 12 | The lower BF in the anterior regions of females and males could play a potential role in the greater stability and longevity of DIs. The OF was lower on the anterior DIs area in both genders due to use implants for aesthetic and less functionally, it may be a concern for good results in the long term. The BF on the posterior regions of males and females was always higher than on the anterior regions that it explained the different occlusion circumstances primarily influenced the amplitude and direction of the BF, and DI entire mandible underwent noticeably increased stress under occlusion. Beside the result of DI stability revealed that it increased in the anterior regions of females and males compared to the posterior regions that it clarified the effective incorporation of the DI into the bone was important for the establishment of the primary and secondary stability.  In addition, the inverse correlation between the OF and DI stability may have been due to the positioning of the posterior DI and the greater masticatory BF, which represented the most important factors influencing the increased OF and decreased DI stability. Therefore, the BF plays a significant role in the long-term success of DIs. Moreover, the understanding of the BF impact, performing a thorough OF analysis, can implementing appropriate treatment strategies are essential for ensuring the stability and longevity of DIs. |
| Other information | |  | | |
| Funding | 22 | Give the source of funding and the role of the funders for the present study and, if applicable, for the original study on which the present article is based |  | Self-funding and the original study on which the present article is based  -Albrektsson T, Zarb G, Worthington P, Eriksson AR. The long-term efficacy of currently used dental implants: a review and proposed criteria of success. Int j oral maxillofac implants. 1986; 1(1):11-25  - Delgado-Ruiz RA, Calvo-Guirado JL, Romanos GE. Effects of occlusal forces on the peri-implant-bone interface stability. J Periodontol 2000. 2019;81(1):179-193.  - Raz P, Meir H, Levartovsky S, Sebaoun A, Beitlitum I. Primary Implant Stability Analysis of Different Dental Implant Connections and Designs—An In Vitro Comparative Study. J Materials. 2022, 15(9): 3072. |

*Give information separately for cases and controls in case-control studies and, if applicable, for exposed and unexposed groups in cohort and cross-sectional studies.

**Note:** An Explanation and Elaboration article discusses each checklist item and gives methodological background and published examples of transparent reporting. The STROBE checklist is best used in conjunction with this article (freely available on the Web sites of PLoS Medicine at http://www.plosmedicine.org/, Annals of Internal Medicine at http://www.annals.org/, and Epidemiology at http://www.epidem.com/). Information on the STROBE Initiative is available at www.strobe-statement.org.
