# Supplementary material for: The impact of bite force on the stability of dental implants
Source: PLoS One. 2026 Jan 20;21(1):e0340355. doi: 10.1371/journal.pone.0340355 (PMC12818635; doi:10.1371/journal.pone.0340355)
Supplement: Attachment — Submitted filename: Response to Reviewers.docx [file pone.0340355.s004.docx]

Thank you for your letter and for the reviewers’ comments concerning our manuscript entitled “. The impact of bite force on the long-term success of dental implants”. Those comments are all valuable and very helpful for revising and improving our paper, as well as the important guiding significance to our research. We have studied comments carefully and have made corrections which we hope meet with approval. The revised portion is marked green and yellow in the paper. The main corrections in the paper and the responses to the reviewer’s comments are as follows:

**Academic Editor of PLOS ONE:** Thank you for providing information to enhance the manuscript about the Journal Requirements

**1-Response to comment:** Please ensure that your manuscript meets PLOS ONE's style requirements

**Response:** Thank you for your comment. I checked and modified the manuscript according to PLOS ONE's style.

**2. Response to comment:** The goal of ensuring long-term data availability to provide all interested researchers

**Response:** Thank you for pointing that out.. We were adding **Data Availability Statement, t**he data that support the findings of this study are available on request from the corresponding author and emails address, Drdhuhahhaziz@gmail.com, dr.rabeemajeed@gmail.com

**Responds to the reviewers’ comments:**

**Reviewer #1:** Thank you for your valuable feedback. I will revise the manuscript to include a clear sentence in green.

**1- Response to comment:** Author has made a good attempt to study. However, the study lacks standardisation

**Response:** Thank you for your comment. I clarify that this study has pre-inserted implant measurements, and these readings depend on digital measurements that gave a standardized measurement of bite force to the natural anterior and posterior teeth, but these readings did not show significant differences from the bit force record after primary loading of a crown on abutment of implant and started function.

Moreover, this study has pre-inserted abutment (initial) stability measurements, and these readings were not significantly different from the stability record after primary loading of the crown on the abutment of the implant and started function.

**2- Response to comment:** The introduction is too long. it should be written around the problem. There should be a problem statement. what was the research question? What were the objectives? Was there any hypothesis?

**Response:** Thank you for your comment. You're correct that the research question, objectives, and hypothesis were written at the end of the introduction and shortening the introduction too.

The research question is whether the BF impact on stability and longevity of DIs. This study aims to explore the effect of the BF on DIs of different genders, to investigate DI stability in both genders, and to assess the correlation between BF and DI stability. The null hypothesis was that the BF could not have a significant impact on the stability and longevity of DIs

**3- Response to comment:** Methodology: The instrument used can only be used to assess the bite force of posteriors. It is not suitable for use in anterior since it does not imitate the anterior force direction on Implants

**Response:** Thank you for pointing that out. I will revise the instrument in Methodology to make it clearer and more explicit with references that the loadstar sensor was used to collect the BF values of posterior and anterior implant areas [28].

**4- Response to comment:** Consider to use how this instrument was used in the methodology. There is no need to explain the mechanism of action of ostell ISQ instrument. Was the study done on Immediately loaded or immediate placement and loading ? please add clarification.

**Response:** Thank you for your comment. The method for monitoring implant stability during healing and immediately loading. The mechanism of action of the Ostell ISQ instrument is very important to give information on how it works and obtain measurements.

**5-Response to comment :** All the instrument and materials shall be mentioned the commercial name, company of manufacture and country of manufacture in brackets as mentioned for first time.

**Response:** Thank you for your suggestion. The Osstell (Integration Diagnostics AB, Göteborg, Sweden).Loadstar sensor, DI-100U, 16-bit load cell interface, Fremont. California. Straumann, Dental Implant System. USA. Medintika, Dental Implant.Germany.

**6-Response to comment:** Consider rewriting the methodology.

**Response:** Thank you for pointing that out. I will revise the i methodology to make it clearer and more explicit. The manuscript methodology was modified according guidelines of the PLOS One journal.

**7-Response to comment:** Limitations of the study and further scope for the study need to be mentioned.

**Response:** Thank you for pointing that out. The limitations and further scope were added at the end of the discussion.

The limitation of this study was that patients who smoked and were hypertensive were not included to avoid bias. The small sample size was included in this study, which caused non-significant differences between study groups, and the increase in subject numbers that required more time for work and follow-up visits throughout the study decision. This study was absence of a control group to compare with because the inclusion criteria of the cohort subjects were not met. The reaction force on the fixed top end of the DI system can serve as a biomechanical reference for future work on DI designs. Further studies of dental implant stability that require follow-up visits for 5-10 years to improve the success of implants.

**8-Response to comment:** Conclusion shall not be descriptive. It has to be written in brief and against the objectives of the study

**Response:** Thank you for your comment. We modified the conclusion to clarify the result of the study and explained these results with the objectives.

**Reviewer #2:** Thank you for your important comments. I will revise the manuscript to include a clear sentence in yellow.

**1. Response to comment:** one issue needs to be addressed, which is the type of opposing occlusion, please mention this and include it in the statistical analysis as a group comparison if some patients had natural occlusions and others had artificial occlusion opposing the implants.

**Response:** Thank you for pointing this out. It was clarified by adding to the inclusion criteria, they had natural occlusions opposing their implants that needed to be inserted. Moreover, the exclusion criteria included: they had missing permanent teeth, an implant, and were restored with a crown of opposing occlusion.

**2. Response to comment:** Also, please correlate your findings with studied implants dimensions

**Response: Thank** you for your suggestion. We correlate the study findings with implant dimensions by adding clear sentences.

Primary stability has also been demonstrated to be influenced by the diameter, surface features, and length of implants. The present study dental implants used with size (4.0*10, 3.5*11, 3.3*10, or 3.8*9) showed high primary and secondary stability. More surface area and a stronger mechanical connection to the tissue around it are provided by rough surfaces of implants [42]. Sandblasted implant surfaces facilitate osteogenesis by increasing osteoblast proliferation and cellular metabolism, according to research conducted in vitro [43,44]. Research has demonstrated the presence of surface response and interaction of cells [45]. Compared to implants with a machining surface, those with acid-etched coatings can achieve a much better bone-to-implant contact in areas with low quality of bone [46]. Experimental evidence has demonstrated that in situations when the amount of bone is limited and implants with diameters below 3.0 millimeters offer adequate initial stability [47]. According to Aparicio et al.'s study on RFA procedures, variables such as supracrestal implant length, abutment length, and upper or lower jaw bone density appear to affect RFA values [48].

The dimensions of implant outcome on stability are consistent with the Raz et al. study's findings, which show that the stability evaluations show greater values for longer implants than for shorter ones, and for densely embedded bone as opposed to softer bone [39].

**3. Response to comment:** please clearly explain the impact of using different thread designs in the tested implants.

**Response:** Thank you for your suggestion.

Implants with a severe thread pattern may improve initial stability [27]. The stability values of tapering implants were consistently higher than those of cylinder implants [29]. These changes involve platform switching and microthreads of DIs used in this study. Compared to non-platform switching designs, platform-switching setups have demonstrated efficient stress performance and reduced the possibility of overloading [49]. The maximum von Mises, compressive, and tensile stresses are reduced when oblique forces are applied to a DI with a platform-switching design compared to a traditional design [50]. The palatal side of the platform and the entire implant surface get a redistribution of the pressures that are moved from the compact bone area to the cancellous bone area [20]. The maximal stresses at the cortical region were lower with platform-switching implants than with conventional implants. Implants with platform switching decreased stress by 40% when subjected to oblique loads and 36% when subjected to axial stresses [21].

After osseointegration and throughout the duration of their use, it is acknowledged that all implants exhibit some degree of loss of bone. According to several claims, the addition of microthreads or "retention grooves" to the implant's neck may help to distribute stress and lessen the amount of bone loss that occurs after installation [51]. In practice, the surgical method and the use of platform switching are linked to the preservation of crestal bone [52]. Additionally, it appears that the progressive thread pattern reduces the crestal bone compression process, hence preventing crestal bone loss [52].

**4. Response to comment:** please have your manuscript edited by a linguistic professional.

**Response:** Thank you for pointing that out. We send the manuscript for proofreading and have a certificate for it.

**Special thanks to you for your good comments.**

We tried our best to improve the manuscript and made some changes in the manuscript. These changes will not influence the content and framework of the paper. And here we highlight the changes marked in yellow and green in the revised paper.

We appreciate for Editors and Reviewers’ warm work, and hope that the correction will meet with approval.

Once again, thank you very much for your comments and suggestions.

We look forward to hearing from you. If you have any queries, please don’t hesitate to contact me anytime at the address below.

Thank you and best regards.

Yours sincerely,
