# Supplementary material for: The impact of bite force on the stability of dental implants
Source: PLoS One. 2026 Jan 20;21(1):e0340355. doi: 10.1371/journal.pone.0340355 (PMC12818635; doi:10.1371/journal.pone.0340355)
Supplement: Attachment — Submitted filename: respons to reviwer.docx [file pone.0340355.s005.docx]

Dear Editors and Reviewers:

Thank you for your letter and for the reviewers’ comments concerning our manuscript entitled “The impact of bite force on the long-term success of dental implants”. Those comments are all valuable and very helpful for revising and improving our paper, as well as the important guiding significance for our research. We have studied comments carefully and have made corrections which we hope meet with approval. The revised portion is marked yellow in the paper.

**Response to the comment of Reviewer 3**

1. Response to comment: 1. Unclear and incomplete results presentation. The data tables do not define the meaning of multiple numbers reported in each cell (e.g., whether they represent mean ± SD, confidence limits, or another metric), preventing independent interpretation.

**Response:** Thank you for your valuable feedback. I will revise the data tables to include a clear sentence in yellow and add the mean ± SD in the tables for further clarification.

This is revised tables:

**Table 1. The Anterior and Posterior Occlusal Forces in Males and Females**

| NO.  of visit | Gender | Anterior Occlusion  mean ± SD | Posterior Occlusion  mean ± SD | Gender | Anterior Occlusion  mean ± SD | Posterior Occlusion mean ± SD |
| --- | --- | --- | --- | --- | --- | --- |
| 1 st | males | 175.3  ± 10.011 | 324  ± 4.475 | females | 154.9  ± 9.573 | 274.2  ± 2.636 |
| 2 nd |  | 172.4  ± 8.212 | 322  ± 6.832 |  | 153.1  ± 8.325 | 272..2  ± 6.221 |
| 3 rd |  | 174.4  ± 6.243 | 323  ± 5.728 |  | 153.4  ± 6.436 | 273..5  ± 3.241 |
| Repeated-Measures ANOVA |  | 21.202 | 39.831 |  | 12.638 | 34.122 |
| *p* value |  | 0.0502 | 0.033 |  | 0.0422 | 0.043 |

Significant (*p*-values ≤.05), non-significant (*p*-values > 05), ± SD: standard deviation.

**Table 2. The Anterior and Posterior Dental Implant Stability in Males and Females**

| NO.  of visit | Gender | Anterior Stability  mean ± SD | Posterior Stability  mean ± SD | Gender | Anterior Stability  mean ± SD | Posterior Stability  mean ± SD |
| --- | --- | --- | --- | --- | --- | --- |
| 1 st | Males | 75.5  ± 4.3 | 72.3  ± 2.11 | Females | 79.9  ± 5.35 | 74.6  ± 3.62 |
| 2^nd^ |  | 72.3  ± 4.112 | 72.8  ± 2.512 |  | 82.3  ± 1.324 | 75.4  ± 3.221 |
| 3 rd |  | 74.1  ± 4.861 | 73.2  ± 2.382 |  | 82.6  ± 1.453 | 76.5  ± 1.734 |
| Repeated-Measures ANOVA |  | 29.017 | 26.023 |  | 36.122 | 31.282 |
| *p* value |  | 0.0532 | 0.044 |  | 0.0518 | 0.037 |

Significant (*p*-values ≤.05), non-significant (*p*-values > 05), ± SD: standard deviation.

**2. Response to comment:** 2. Figures suggest non-linear patterns, yet only linear fits are superimposed without justification, obscuring true relationships.

**Response:** Thank you for your suggestion. I was explaining of mean value levels of Occlusal Force and Dental Implant Stability in both genders in Figures 2 and 3. The scatter plot should be explicitly stated for clarity.

This is revised:

Occlusal Force of Males

occlusal Forces

| Number of visits | Anterior Occlusion | Posterior Occlusion |
| --- | --- | --- |
| 1 st | 175.3 | 324 |
|  | 0 |  |
| 2 nd | 172.4 | 322 |
| 3 rd | 174.4 | 323 |

(a)

Occlusal Forces of Females

Occlusal Forces

| Number of visites | Anterior Occlusion | Posterior Occlusion |
| --- | --- | --- |
| 1 st | 154.9 | 274.2 |
| 2 nd | 153.1 | 272.2 |
| 3 rd | 153.4 | 273.5 |

(b)

Figure 2. The mean value differences of anterior occlusal forces and posterior occlusal forces in males (a); The mean value differences of anterior occlusal forces and posterior occlusal forces in females (b).

Implant Stability of Males

Stability

| Number of visites | Anterior Stability | Posterior Stability |
| --- | --- | --- |
| 1 st | 75.5 | 72.3 |
| 2nd | 72.3 | 72.8 |
| 3 rd | 74.1 | 73.2 |

**(a)**

Dental Implant Stability of Females

Stability

| Number of visites | Anterior Stability | Posterior Stability |
| --- | --- | --- |
| 1 st | 79.9 | 74.6 |
| 2nd | 82.3 | 75.4 |
| 3 rd | 82.6 | 76.5 |

**(b)**

Figure 3. The mean value differences of anterior implant stability and posterior implant stability in males (a); The mean value differences of anterior implant stability and posterior implant stability in females (b).

3. Response to comment: 3. Inappropriate and insufficient statistical methodology. Normality‐ or variance-homogeneity checks were not reported, so the suitability of parametric tests is unverified.

Response: Thank you for your comment. I was adding detailed reporting of how these analyses were conducted in the revision. I will rewrite the statistical analyses methodology of normality, another test for comparisons between more than two groups, and *P* < 0.05 was considered statistically significant.

This is revised:

Statistical Analyses

Descriptive statistics through GraphPad® Prism 9.5.1 were used to summarize the patient demographics, BF measurements, and DI stability measurements. The normality of distribution was checked by the Shapiro–Wilk and D’Agostino’s tests. The continuous variables were expressed as the mean standard deviation (±), while the categorical variables were presented as frequencies and percentages. Comparative analyses, such as an analysis of variance (Repeated-Measures ANOVA), were performed to assess differences in the BF and DI stability in males and females at the anterior and posterior areas of the jaws among three postoperative time points. For comparisons between more than two groups, Tukey’s honestly significant difference Post Hoc tests were used. A Pearson correlation was done to investigate the relationship between the BF and DI stability, and *P* < 0.05 was considered statistically significant. If any patient in this study missed a follow-up visit, the author would call the patient to revisit with a new appointment.

4- Response to comment: 4. A one-way ANOVA was applied to compare first, second, and third observations, although a repeated-measures ANOVA (or a mixed model) is required for dependent data.

Response: Thank you for your question. I was writing a new analysis of these data, depending on repeated-measures ANOVA and p-value in Tables 1 and 2 in revision.

This is revised:

. **Table 1. The Anterior & Posterior Occlusal Forces in Males & Females**

| NO.  of visit | Gender | Anterior Occlusion  mean ± SD | Posterior Occlusion  mean ± SD | Gender | Anterior Occlusion  mean ± SD | Posterior Occlusion mean ± SD |
| --- | --- | --- | --- | --- | --- | --- |
| 1 st | males | 175.3  ± 10.011 | 324  ± 4.475 | females | 154.9  ± 9.573 | 274.2  ± 2.636 |
| 2 nd |  | 172.4  ± 8.212 | 322  ± 6.832 |  | 153.1  ± 8.325 | 272..2  ± 6.221 |
| 3 rd |  | 174.4  ± 6.243 | 323  ± 5.728 |  | 153.4  ± 6.436 | 273..5  ± 3.241 |
| Repeated-Measures ANOVA |  | 21.202 | 39.831 |  | 12.638 | 34.122 |
| *p* value |  | 0.0502 | 0.033 |  | 0.0422 | 0.043 |

Significant (*p*-values ≤.05), non-significant (*p*-values > 05), ± SD: standard deviation.

**Table 2. The Anterior and Posterior Dental Implant Stability in Males and Females**

| NO.  of visit | Gender | Anterior Stability  mean ± SD | Posterior Stability  mean ± SD | Gender | Anterior Stability  mean ± SD | Posterior Stability  mean ± SD |
| --- | --- | --- | --- | --- | --- | --- |
| 1 st | Males | 75.5  ± 4.3 | 72.3  ± 2.11 | Females | 79.9  ± 5.35 | 74.6  ± 3.62 |
| 2^nd^ |  | 72.3  ± 4.112 | 72.8  ± 2.512 |  | 82.3  ± 1.324 | 75.4  ± 3.221 |
| 3 rd |  | 74.1  ± 4.861 | 73.2  ± 2.382 |  | 82.6  ± 1.453 | 76.5  ± 1.734 |
| Repeated-Measures ANOVA |  | 29.017 | 26.023 |  | 36.122 | 31.282 |
| *p* value |  | 0.0532 | 0.044 |  | 0.0518 | 0.037 |

Significant (*p*-values ≤.05), non-significant (*p*-values > 05), ± SD: standard deviation.

**5- Response to comment**: 5. Correlations were assessed with simple linear regression despite visual evidence of non-linearity and the absence of diagnostic plots or residual analyses.

**Response：** Thank you for this suggestion. A Pearson correlation was done to investigate the relationship between the BF and DI stability in both genders Table 4. As well as the relationship was illustrated in a diagnostic scatter plot between the OF and DI stability.

This is revised:

| Anterior Implant stability in males | Anterior OF in males |
| --- | --- |
| 89 | 180 |
| 88 | 170 |
| 78 | 176 |
| 93 | 185 |
| 87 | 165 |
| 94 | 173 |
| 94 | 185 |
| 96 | 177 |
| 88 | 187 |
| 86 | 155 |
| 76 | 180 |
| 78 | 170 |
| 89 | 176 |
| 82 | 185 |
| 83 | 165 |
| 95 | 173 |
| 86 | 185 |
| 93 | 177 |
| 88 | 187 |
| 85 | 155 |

**(a)**

| Posterior Implant stability in males | Posterior OF in males |
| --- | --- |
| 73 | 260 |
| 74 | 300 |
| 78 | 330 |
| 73 | 400 |
| 72 | 340 |
| 77 | 290 |
| 68 | 390 |
| 67 | 320 |
| 78 | 340 |
| 69 | 270 |
| 73 | 260 |
| 71 | 300 |
| 75 | 330 |
| 76 | 400 |
| 74 | 340 |
| 65 | 290 |
| 64 | 390 |
| 66 | 320 |
| 75 | 340 |
| 80 | 270 |

**(b)**

| Anterior Implant Stability in females | Anterior OF in females |
| --- | --- |
| 88 | 160 |
| 89 | 157 |
| 85 | 166 |
| 79 | 170 |
| 93 | 161 |
| 94 | 152 |
| 94 | 145 |
| 96 | 140 |
| 88 | 151 |
| 86 | 147 |
| 76 | 160 |
| 78 | 157 |
| 89 | 166 |
| 82 | 170 |
| 83 | 161 |
| 82 | 152 |
| 86 | 145 |
| 77 | 140 |
| 79 | 151 |
| 85 | 147 |

**(c)**

| Poterior Implant Stability in females | Posterior OF in females |
| --- | --- |
| 78 | 280 |
| 76 | 240 |
| 66 | 300 |
| 71 | 310 |
| 73 | 290 |
| 66 | 280 |
| 69 | 295 |
| 71 | 250 |
| 74 | 266 |
| 69 | 231 |
| 73 | 280 |
| 64 | 240 |
| 76 | 300 |
| 71 | 310 |
| 68 | 290 |
| 67 | 280 |
| 68 | 295 |
| 69 | 250 |
| 63 | 266 |
| 68 | 231 |

**(d)**

| Implant Stability in Males | OF in Males |
| --- | --- |
| 89 | 180 |
| 88 | 170 |
| 78 | 176 |
| 93 | 185 |
| 87 | 165 |
| 94 | 173 |
| 94 | 185 |
| 96 | 177 |
| 88 | 187 |
| 86 | 155 |
| 76 | 180 |
| 78 | 170 |
| 89 | 176 |
| 82 | 185 |
| 83 | 165 |
| 95 | 173 |
| 86 | 185 |
| 93 | 177 |
| 88 | 187 |
| 85 | 155 |
| 73 | 260 |
| 74 | 300 |
| 78 | 330 |
| 73 | 400 |
| 72 | 340 |
| 77 | 290 |
| 68 | 390 |
| 67 | 320 |
| 78 | 340 |
| 69 | 270 |
| 73 | 260 |
| 71 | 300 |
| 75 | 330 |
| 76 | 400 |
| 74 | 340 |
| 65 | 290 |
| 64 | 390 |
| 66 | 320 |
| 75 | 340 |
| 80 | 270 |

**(e)**

| Implant Stability in Females | OF in Females |
| --- | --- |
| 88 | 160 |
| 89 | 157 |
| 85 | 166 |
| 79 | 170 |
| 93 | 161 |
| 94 | 152 |
| 94 | 145 |
| 96 | 140 |
| 88 | 151 |
| 86 | 147 |
| 76 | 160 |
| 78 | 157 |
| 89 | 166 |
| 82 | 170 |
| 83 | 161 |
| 82 | 152 |
| 86 | 145 |
| 77 | 140 |
| 79 | 151 |
| 85 | 147 |
| 78 | 280 |
| 76 | 240 |
| 66 | 300 |
| 71 | 310 |
| 73 | 290 |
| 66 | 280 |
| 69 | 295 |
| 71 | 250 |
| 74 | 266 |
| 69 | 231 |
| 73 | 280 |
| 64 | 240 |
| 76 | 300 |
| 71 | 310 |
| 68 | 290 |
| 67 | 280 |
| 68 | 295 |
| 69 | 250 |
| 63 | 266 |
| 68 | 231 |

**(f)**

| Implant Stability | OF |
| --- | --- |
| 89 | 180 |
| 88 | 170 |
| 78 | 176 |
| 93 | 185 |
| 87 | 165 |
| 94 | 173 |
| 94 | 185 |
| 96 | 177 |
| 88 | 187 |
| 86 | 155 |
| 76 | 180 |
| 78 | 170 |
| 89 | 176 |
| 82 | 185 |
| 83 | 165 |
| 95 | 173 |
| 86 | 185 |
| 93 | 177 |
| 88 | 187 |
| 85 | 155 |
| 73 | 260 |
| 74 | 300 |
| 78 | 330 |
| 73 | 400 |
| 72 | 340 |
| 77 | 290 |
| 68 | 390 |
| 67 | 320 |
| 78 | 340 |
| 69 | 270 |
| 73 | 260 |
| 71 | 300 |
| 75 | 330 |
| 76 | 400 |
| 74 | 340 |
| 65 | 290 |
| 64 | 390 |
| 66 | 320 |
| 75 | 340 |
| 80 | 270 |
| 88 | 160 |
| 89 | 157 |
| 85 | 166 |
| 79 | 170 |
| 93 | 161 |
| 94 | 152 |
| 94 | 145 |
| 96 | 140 |
| 88 | 151 |
| 86 | 147 |
| 76 | 160 |
| 78 | 157 |
| 89 | 166 |
| 82 | 170 |
| 83 | 161 |
| 82 | 152 |
| 86 | 145 |
| 77 | 140 |
| 79 | 151 |
| 85 | 147 |
| 78 | 280 |
| 76 | 240 |
| 66 | 300 |
| 71 | 310 |
| 73 | 290 |
| 66 | 280 |
| 69 | 295 |
| 71 | 250 |
| 74 | 266 |
| 69 | 231 |
| 73 | 280 |
| 64 | 240 |
| 76 | 300 |
| 71 | 310 |
| 68 | 290 |
| 67 | 280 |
| 68 | 295 |
| 69 | 250 |
| 63 | 266 |
| 68 | 231 |

**(g)**

Figure 4 Scatter plot showing the relationship between Anterior OF in males and Anterior DIs stability in males (a); Scatter plot showing the relationship between Posterior OF in males and Posterior DIs stability in males (b); Scatter plot showing the relationship between Anterior OF in females and Anterior DIs stability in females (c ); Scatter plot showing the relationship between Posterior OF in females and Posterior DIs stability in females (d); Scatter plot showing the relationship between OF in males and DIs stability in males (e); Scatter plot showing the relationship between OF and DIs stability in females (f); and Scatter plot showing the relationship between OF and DIs stability (g).

6- Response to comment: 6. Mismatch between stated aims and executed analyses. Also, not static the hypothesis as suggested.

**Response:** Thank you for your valuable feedback. I will revise the stated aims and executed analyses to include a clear sentence for further clarification.

This is revised:

This study aims to explore the effect of the BF on DIs of different genders, to investigate DI stability in both genders, and to assess the correlation between BF and DI stability in both genders. The null hypothesis is that the BF could not have a significant impact on the stability of DIs. As well as the relationship was illustrated in a diagnostic scatter plot between the OF and DI stability in both genders.

7- Response to comment: The aim explicitly includes gender comparison (“…effect of BF on DIs of different genders…”), yet no direct gender stratification or interaction term appears in the statistical model.

**Response:** Thank you for pointing that out. I was adding details of the test for comparisons between more than two groups, Tukey’s honestly significant difference Post Hoc tests were used in revision in Table 3.

**Table 3. Intergroup Comparisons of Mean Occlusal Force and Stability in both genders.**

| Variable | Groups | Groups | Tukey’s HSD Post Hoc test | p |
| --- | --- | --- | --- | --- |
| Occlusal Force | Anterior OF in males | Posterior OF in males | 22.490 | 0.000 |
|  |  | Anterior OF in females | 17.349 | 0.003 |
|  |  | Posterior OF in females | 38.205 | 0.001 |
|  | Posterior OF in males | Anterior OF in females | 24.859 | 0.041 |
|  |  | Posterior OF in females | 15.715 | 0.027 |
|  | Anterior OF in females | Posterior OF in females | 30.856 | 0.002 |
| Stability | Anterior DIs stability in males | Posterior DIs stability in males | 29.268 | 0.002 |
|  |  | Anterior DIs stability in females | 18.824 | 0.003 |
|  |  | Posterior DIs stability in females | 34.179 | 0.000 |
|  | Posterior DIs stability in males | Anterior DIs stability in females | 36.557 | 0.001 |
|  |  | Posterior DIs stability in females | 17.911 | 0.000 |
|  | Anterior DIs stability in females | Posterior DIs stability in females | 43.355 | 0.004 |

Significant at (*p* <0.05); Tukey’s HSD Post Hoc test: Tukey (honestly significant differences

8- Response to comment: 8. Unsupported and overstated conclusions. The conclusion section reiterates some findings (results) and speculates on gender differences without empirical support.

Response: Thank you for your question. I revised the conclusion, which comprises a clear sentence for further clarification, depending on the strength of correlation between OF and DIs stability in both genders, and the potential impact in both genders.

This is revised:

## **Conclusion**

The significant correlation between BF and DI stability in both genders plays a noteworthy role in the long-term success of DIs. The significantly lower BF in the anterior regions of females and males may have an impact role in the significantly greater stability of DIs. The OF was lower on the anterior DIs area in both genders due to the use of implants for aesthetic purposes, and less functionally. Besides, the result of DI stability revealed that it increased in the anterior regions of females and males compared to the posterior regions, it clarified that the effective incorporation of the DI into the bone was important for the establishment of the primary and secondary stability.

In addition, the inverse relationship between the OF and DI stability may have been due to the positioning of the posterior DI and the greater masticatory BF, which represented the most important factors influencing the increased OF and decreased DI stability. Moreover, the understanding of the BF impact, performing a thorough OF analysis, can implementing appropriate treatment strategies are essential for ensuring the stability and longevity of DIs.

9- Response to comment: 9. Several claims (e.g., superiority of one gender in long-term stability) extend beyond the study’s data and timeframe.

**Response:** Thank you for your question. This study found a significant correlation between BF and DI stability in both genders. The significantly lower mean BF in the anterior regions of females and males after delivery of dental implant restoration could play a potential role in the significantly greater mean stability of DIs during the one half year following insertion, with three follow-up visits. The significant correlation between BF and DI stability in both genders plays a noteworthy role in the long-term success of DIs.

**Special thanks to you for your good comments.**

We tried our best to improve the correction of the manuscript and made some changes in the revision manuscript. These changes will not influence the content and framework of the paper. And here we highlight the changes marked in yellow in the revised paper.

We appreciate for Editors and Reviewers’ warm work earnestly, and hope that the correction will meet with approval.

Once again, thank you very much for your comments and suggestions.

We look forward to hearing from you. If you have any queries, please don’t hesitate to contact me anytime at the address below.

Thank you and best regards.

Yours sincerely,
