# Supplementary material for: The impact of bite force on the stability of dental implants
Source: PLoS One. 2026 Jan 20;21(1):e0340355. doi: 10.1371/journal.pone.0340355 (PMC12818635; doi:10.1371/journal.pone.0340355)
Supplement: Attachment — Submitted filename: respons to reviewers.docx [file pone.0340355.s006.docx]

Dear Editors and Reviewers:

Thank you for your letter and for the reviewers’ comments concerning our manuscript entitled “The impact of bite force on the stability of dental implants”. Those comments are all valuable and very helpful for revising and improving our paper, as well as the important guiding significance for our research. We have studied comments carefully and have made corrections which we hope will meet with approval. The revised portion is marked green in the paper.

**Response to the comment of Reviewer 4**

**1. Response to comment: 1. Methods — participants**

- Clarify inclusion/exclusion precisely and remove contradictory phrasing.

- Provide a flow diagram (enrolment → allocation → follow-up → analysis) with numbers lost to follow‑up and reasons.

**Response:** Thank you for your valuable feedback. I will revise the inclusion/exclusion precisely and remove contradictory phrasing to include a clear sentence in green and add a flow diagram.

**This is revised**

The inclusion criteria were male and female patients aged 25-40 years, who had sufficient bone volume for DI placements instead missing number of natural teeth and had natural occlusions, opposed natural teeth should be presented to implants that needed to be inserted, understood and signed an informed consent form, and were followed up at postoperative visits. Patients with a history of systemic diseases affecting bone metabolism, periodontitis, cigarette smoking, or drinking alcohol were excluded from the study. Moreover, the exclusion criteria included: they had missing permanent teeth which replaced by an implant, and a restoration with a crown of opposing, delayed loading, and parafunctional habits.

**2. Response to comment: 2. Methods — sample size**

- Report the exact G*Power inputs (effect size f for d, α, power, correlation among repeated measures, number of groups/time points) and how 80 participants were chosen.

Response: Thank you for your suggestion. I was explaining sample size calculation by using G*Power inputs (effect size f, α, power, correlation among repeated measures, number of groups/time points) and how 80 participants were chosen. It depended on the pilot study resulting of 12 patients with DIs placed, three in each group (3 patients placed DIs for anterior regions of male, 3 patients placed DIs for posterior regions of male, 3 patients placed DIs for anterior regions of female and 3 patients placed DIs for posterior regions of female) that BF and ISQ measured during three visits interval (first visit during the day of insertion of the crown on the abutment of the implant (immediately loading), second visit at six months following insertion, and third visit after 18 months following insertion. Then these data were analyzed and resulted in a significant effect size of F at 0.4, and four groups. The total number of participants was around 80 to avoid patient dropouts, and these were divided into four groups.

**This is revised**

Sample Size Power Analysis

The sample size, calculated by G power, comprised 18 individuals in each group at a power of 80, an α probability of 0.05 that it depended on the pilot study resulting of 12 patients with DIs placed, three in each group (3 patients placed DIs for anterior regions of male, 3 patients placed DIs for posterior regions of male, 3 patients placed DIs for anterior regions of female and 3 patients placed DIs for posterior regions of female) that BF and ISQ measured during three visits interval (first visit during the day of insertion of the crown on the abutment of the implant (immediately loading), second visit at six months following insertion, and third visit after 18 months following insertion. Then these data were analysed and resulted in a significant effect size of F at 0.4, and four groups. The total number of participants was around 80 to avoid patient dropouts, and these were divided into four groups. 80 individuals of both genders who had lost some teeth and needed DIs for the anterior and posterior regions of the jaws.

**3. Response to comment. Methods — statistical analysis (revise and expand)**

A- State data structure: subjects measured at 3 time points; BF and ISQ measured at anterior/posterior sites; sex as a between-subjects factor.

B- Use an appropriate repeated-measures model: mixed-effects model (preferred) or two-way repeated-measures ANOVA including time (within), site (within), and sex (between), and their interactions.

C- Report checks: normality (Shapiro-Wilk) on residuals, homogeneity, sphericity (Mauchly), and handling of violations (Greenhouse-Geisser or mixed model). Show test statistics and p-values.

D- For correlations, use within-subject paired analyses (e.g., compute BF–ISQ correlation across measurements per subject and use mixed-effects regression to model ISQ ~ BF + time + site + sex + random intercept(subject)). Report 95% CIs, p-values, and model diagnostics (residuals, influence).

E- Correct for multiple comparisons or justify not doing so.

**Response:** Thank you for your comment.

A-I was adding the state data structure in the data collection part of the article.

B-I was adding detailed reporting of repeated-measures model in two-way repeated-measures ANOVA, including time (within), site (within), and sex (between), and their interactions. These analyses were conducted in the revision of Table 4.

C- I was explaining how to check the normality (Shapiro-Wilk) on residuals and homogeneity. The sphericity (Mauchly) and handling of violations (Greenhouse-Geisser on mixed model) were analysed in Table 5.

D-I was added to table 8 using mixed-effects regression to model ISQ ~ BF + time + site + sex + random intercept (subject) and 95% CIs, p-values

E – I corrected the multiple comparisons with the Bonferroni test in bite force and stability after justifying other factors.

This is revised

A-Data Collection

State data structure, subjects measured at 3 time points; BF and ISQ measured at anterior/posterior sites; sex as a between-subjects factor. Relevant clinical information, such as the patient’s characteristics after placement with a DI by a specialist, medical background, X-ray pictures, and scans of the inside of the mouth, was obtained from the computerised medical records and documents of the patient. A Loadstar™ sensor was used to collect the BF values of posterior and anterior implant areas [28]. This sensor offers various force-measuring solutions with updated data rates of up to 50 KHz, making it suitable for applications that require the BF to be noted soon after the operation and during consultation intervals. Regular monitoring of the BF and DI stability was crucial in the post-implantation phase. Loadstar™ sensor records were utilised to assess the OF and identify any abnormalities or imbalances. While the Osstell Implant Stability Quotient (ISQ) technology was used to evaluate the DI stability by examining the resonance frequency of the DI. The patients were also educated on proper oral hygiene practices, including avoiding excessive BF on the DI, maintaining regular dental visits, and promptly addressing any signs of discomfort or changes in the bite. The data availability was presented in supporting information files.

B- Table 4. Results of the two-way ANOVA of all groups for the implant occlusal forces and stability values between the time, locations of the implants, and gender.

| Value |  | 1^st^ | 2^nd^ | 3^rd^ |
| --- | --- | --- | --- | --- |
| Bite forces(N) | Time | 0.750 | 0.051* | 0.075 |
|  | locations of the implants | 0.657 | 0.534 | 0.042* |
|  | Time **×** Location | 0.134 | 0.016 * | 0.042 * |
|  | gender | 0.119 | 0.052 | 0.157 |
|  | gender × Time | 0.250 | 0.007 * | 0.870 |
|  | gender × Location | 0.720 | 0.321 | 0.520 |
| stabilityISQ | Time | 0.923 | 0.809 | 0.162 |
|  | locations of the implants | 0.185 | 0.436 | 0.137 |
|  | Time × Location | 0.977 | 0.811 | 0.516 |
|  | gender | 0.170 | 0.162 | 0.259 |
|  | gender × Time | 0.186 | 0.044 * | 0.199 |
|  | gender × Location | 0.472 | 0.199 | 0.289 |

* denotes a significant difference, with p < 0.05.

C-. The Shapiro-Wilk test was used to detect whether the quantitative data were normally distributed. The normality of distribution (parametric)was significantly result by used the Shapiro–Wilk. The parametric data was analyzed.

Table 5. Results of the sphericity (Mauchly), and handling of violations (Greenhouse-Geisser

Table 5. Results of the sphericity (Mauchly), and handling of violations (Greenhouse-Geisser

| Residual variables | Influence variables | 1 First visit | 2 Second visit | 3 Third visit | Mauchly | Greenhouse-Geisser |
| --- | --- | --- | --- | --- | --- | --- |
| Bite forces(N) | Time | 0.750 | 0.051* | 0.075 | 0.071 | 0.052 |
|  | locations of the implants | 0.657 | 0.534 | 0.042* | 0.018* | 0.001 |
|  | Time **×** Location | 0.134 | 0.016 * | 0.042 * | 0.001* | 0.000 |
|  | gender | 0.119 | 0.052 | 0.157 | 0.093 | 0.076 |
|  | gender × Time | 0.250 | 0.007 * | 0.870 | 0.024* | 0.001* |
|  | gender × Location | 0.072 | 0.032* | 0.052* | 0.001* | 0.000* |
| Stability ISQ | Time | 0.923 | 0.809 | 0.162 | 0.063 | 0.099 |
|  | locations of the implants | 0.0185 | 0.0436 | 0.0137 | 0.0591* | 0.003* |
|  | Time × Location | 0.097 | 0.011* | 0.051* | 0.022* | 0.000* |
|  | gender | 0.170 | 0.162 | 0.259 | 0.001* | 0.001* |
|  | gender × Time | 0.186 | 0.044 * | 0.191 | 0.007* | 0.000* |
|  | gender × Location | 0.472 | 0.019* | 0.028* | 0.004* | 0.000* |

* denotes a significant difference, with p < 0.05.

D- Table 8. Differences in regression coefficients (b) for BF and implant ISQ between the Time, Location of Implants, and Gender. b illustrated the mean percentage change in one unit of influencing variables (Time, Location of Implants, and Gender) based on the change in BF and implant ISQ. Significant differences in the regression coefficients (b) are denoted by * (p < 0.05). The term ratio reflects the quotient of bISQ / bBF.

| Variables | |  | 1 First visit |  | 2 Second visit |  | 3 Third visit |
| --- | --- | --- | --- | --- | --- | --- | --- |
| Influencing variables | Residual  varibles | b (%) | 95% CI | b (%) | 95% CI | b (%) | 95% CI |
| Time | BF | 12.4 * | (10.7-13.8) | 16.1* | 4.6 10.2 | 19.8* | 13.5 17.9 |
|  | ISQ | 39.3 * | (25.5-56.9) | 49.2 | 66.7 107.6 | 58.4* | 70.2 97.1 |
|  | Ratio | 3.1 |  | 3.0 |  | 2.9 |  |
| Location of Implants | BF | 19.4 * | (12.6- 17.6) | 22.8* | 5.5 10.1 | 24.6 | 3.9 5.8 |
|  | ISQ | 40.8 * | (79.2- 86.0) | 56.2* | 127.3 83.2 | 66.4* | 44.0 58.5 |
|  | Ratio | 2.1 |  | 2.4 |  | 2.6 |  |
| Gender | BF | 16.4 * | 12.6 17.6 | 24.5* | 13.5 17.9 | 30.2* | 14.4 18.4 |
|  | ISQ | 62.5* | 79.2 86.0 | 78.3 | 82.2 97.1 | 88.1* | 53.7 102.3 |
|  | Ratio | 3.8 |  | 3.1 |  | 2.9 |  |

* denotes a significant difference, with p < 0.05.

E- **Table 6. Intergroup Comparisons of Mean Occlusal Force and Stability in both genders. the Means Comparison tab, select the Bonferroni test for bite force and stability**

| Variable | Groups | Groups | Bonferroni test | p value |
| --- | --- | --- | --- | --- |
| Bte Force | Anterior BF in males | Posterior BF in males | 23.84 | 0.008 |
|  |  | Anterior BF in females | 11.922 | 0.042 |
|  |  | Posterior BF in females | 33.42 | 0.055 |
|  | Posterior BF in males | Anterior OF in females | 39.21 | 0.057 |
|  |  | Posterior BF in females | 18.22 | 0.013 |
|  | Anterior BF in females | Posterior BF in females | 28.63 | 0.034 |
| Stability | Anterior DIs stability in males | Posterior DIs stability in males | 32.08 | 0.011 |
|  |  | Anterior DIs stability in females | 21.46 | 0.028 |
|  |  | Posterior DIs stability in females | 28.36 | 0.046 |
|  | Posterior DIs stability in males | Anterior DIs stability in females | 31.68 | 0.003 |
|  |  | Posterior DIs stability in females | 19.22 | 0.005 |
|  | Anterior DIs stability in females | Posterior DIs stability in females | 39.114 | 0.001 |

Significant at (*p* <0.05); Bonferroni test.

**4- Response to comment:4- Reporting of results**

A- Clean all tables: each cell must show mean ± SD (or median [IQR] if nonparametric). Remove typographical artifacts and ensure consistent units (N for force, ISQ units for stability).

B- Report sample sizes for each stratum/time point (n per cell).

C- Report effect sizes (ANOVA; regression coefficients with 95% CI; r and adjusted R^2 for correlations/regressions).

**Response:** Thank you for your question.

A-I was the clear mean ± SD of parametric data in cells of Table 2,3. I added the consistent units (N for force, ISQ units for stability).

B-I was reporting the sample sizes for each stratum/time point (n per cell) in table 2,3.

C- I was writing a new analysis of these data, depending on ANOVA, regression coefficients with 95% CI; r and adjusted R^2 for correlations/regressions.

**5- Response to comment**: 5. Confounder adjustment: Include implant-level and patient-level covariates in models: implant length/diameter, jaw (maxilla/mandible), bone quality rating (if available), immediate vs delayed loading, opposing dentition, age, and parafunctional habits.

**Response：** Thank you for this suggestion. I was clarifying the details of implant-level and patient-level covariates in models in Table 1.

**This is revised**

Table 1. Demographic data.

|  | DIs for the anterior regions of males | DIs for the posterior regions of males | DIs for the anterior regions of females | DIs for the posterior regions of females | p value |
| --- | --- | --- | --- | --- | --- |
| Number of patients (total) | 20 | 20 | 20 | 20 | 0.11 |
| Age | 35 | 37 | 36 | 34 | 0.18 |
| Gender | 20 | 20 | 20 | 20 | 0.21 |
| Number of implants (total) | 45 | 76 | 43 | 71 | 0.79 |
| Implant diameter |  |  |  |  |  |
| 3.3 mm | 8 | 10 | 7 | 12 | 0.122 |
| 3.5 mm | 19 | 22 | 23 | 21 | 0.084 |
| 3.8 mm | 14 | 24 | 11 | 30 | 0.056 |
| 4 mm | 4 | 20 | 2 | 8 | 0.077 |
| Length |  |  |  |  |  |
| 9 mm | 13 | 49 | 15 | 42 | 0.095 |
| 10 mm | 25 | 22 | 19 | 23 | 0.082 |
| 11 mm | 7 | 5 | 9 | 6 | 0.112 |

Significant (*p*-values ≤.05), non-significant (*p*-values > 05

**6- Response to comment: 6. Hypothesis framing and interpretation**

- Restate primary and secondary hypotheses, predefined primary outcome (e.g., change in ISQ at 12 months), and avoid causal language; discuss directionality and limits of observational data.

- Tone down claims about long-term success unless long-term (≥5 years) data exist.

**Response：** Thank you for this suggestion. I was corrected primary outcome, secondary outcome, and hypotheses.

**This is revised**

The hypothesis is that there was a change in BF and ISQ of DIs during 18 months, followed insertion of the crown on the abutment of the implant. The null hypothesis is that there was no change in BF and ISQ of DIs during 18 months, following the insertion of the crown on the abutment of the implant.

Outcome Measures

The main objective of the study was to determine the relationship between the BF and the stability of DIs during the 18 months that followed. The secondary outcomes included influencing time, gender, and the DI location in the jaws on the BF and the DI stability.

**7-** Response to comment: 7. Figures

- Improve figure clarity

Response: Thank you for your valuable feedback. I added Figure 2. The CONSORT 2010 flow diagram, and clear other figures.

**8- Response to comment: 8. Tables of implants and procedural details**

Provide a table summarizing implant characteristics (brand, diameter, length, surface, site distribution) and surgical/prosthetic protocol (immediate loading protocol details, abutment type, whether platform switching was used).

**Response：** Thank you for this suggestion. I added Table 1 summarizing implant characteristics (diameter, length, and number of implants in each size and group). Further surgical procedure (immediate loading protocol details.

**This is revised**

Table 1. Demographic data.

|  | DIs for the anterior regions of males | DIs for the posterior regions of males | DIs for the anterior regions of females | DIs for the posterior regions of females | p value |
| --- | --- | --- | --- | --- | --- |
| Number of patients (total) | 20 | 20 | 20 | 20 | 0.11 |
| Age | 35 | 37 | 36 | 34 | 0.18 |
| Gender | 20 | 20 | 20 | 20 | 0.21 |
| Number of implants (total) | 45 | 76 | 43 | 71 | 0.79 |
| Implant diameter |  |  |  |  |  |
| 3.3 mm | 8 | 10 | 7 | 12 | 0.122 |
| 3.5 mm | 19 | 22 | 23 | 21 | 0.084 |
| 3.8 mm | 14 | 24 | 11 | 30 | 0.056 |
| 4 mm | 4 | 20 | 2 | 8 | 0.077 |
| Length |  |  |  |  |  |
| 9 mm | 13 | 49 | 15 | 42 | 0.095 |
| 10 mm | 25 | 22 | 19 | 23 | 0.082 |
| 11 mm | 7 | 5 | 9 | 6 | 0.112 |

Surgical Procedure

The implants distributed were placed in the anterior and posterior regions of the jaws (Table 1). Following dental cone-beam computed tomography (CBCT), the quantity and quality of bone were assessed, and a stent was created for implantation at the proper location for each patient. No bone augmentation treatment was used throughout the implant placement process. As instructed by the manufacturer, the location was drilled using a point Lindemann drill first, then surgical drills. A skilled researcher meticulously drilled every dental implant bed at a consistent length and angles in order to produce comparable insertion torque values (ITV) of about 35 Ncm amongst the implants. A drilling machine specifically made for implant surgery was used for measuring ITV as much as 35 Ncm at around 20 rpm and 8 Hz. The drill unit's handpiece was the only tool used to put each implant. Following surgery, CBCT was utilized to assess the integrity of the osseous tissue surrounding the implant [28].

**9- Response to comment: 9. Data and ethics**

A- Confirm anonymization procedures for shared data and provide clear instructions for accessing supporting files; ensure consent wording matches data sharing.

B- Include ethics committee name, reference number, and date of approval as per journal requirements.

**Response:** Thank you for your valuable feedback.

A-I was confirming anonymization procedures for shared data and providing clear instructions for accessing supporting files; ensured consent wording matches data sharing.

B- I added the ethics committee name, reference number, and date of approval as per journal requirements.

**This is revised**

A-Data Availability Statement

The data that support the findings of this study are available in the Supporting Information files. This project contains the following underlying data the data contains the recording of bite force of the male and female participants after the insertion of dental implants for the anterior and posterior regions of the jaws. The dental implants' stability was monitored at the anterior and posterior regions of both genders' jaws. For determining the impact of the BF on the stability of dental implants. Data are available under the terms CC0. All participants entered the study after they received full information about the nature, aims, processes of the study, and publication of raw data (without containing the name of participants) before signing an informed written consent form.

B- All procedures performed in this study involving human participants were in accordance with the Declaration of Helsinki and its later amendments for human research. The study was conducted in accordance with and approved by the Ethics Committee of the College of Dentistry, University of Al-Ameed, Iraq (#52017, 20 February/2021). Written informed consent was obtained from all subjects and/or their legal guardian (s), where all adult participants entered the study after they received full information about the nature, aims, processes of the study, data sharing, the anonymization of participants in deposited data and publication of raw data (without containing the name of participants) before signing an informed written Consent form.

**10- Response to comment: 10. Language and editing**

- Correct typographical errors and awkward phrasing throughout; standardize use of terms: bite force (BF), occlusal force (OF) — choose one term and use consistently.

**Response:** Thank you for pointing that out. I was editing the incorrect words and standardizing BF.

**Statistical issues explained and required changes.**

A-Model selection: Repeated observations per subject violate independence; simple one-way ANOVA or separate ANOVAs per group are inappropriate. Use linear mixed-effects models with random intercept (subject) and, if warranted, random slope (time) to account for within-subject correlation and unequal spacing, and include interaction terms (time × sex, time × site, BF × site).

**Response:** Thank you for your valuable feedback. I will revise the data tables to include a clear sentence in green and add the tables4,5 for further clarification. Table 4. Results of the two-way ANOVA of all groups for the implant bite forces and stability values between the time, locations of the implants, and gender with mixed-effects models. Adding the interaction terms (time × sex, time × site, BF × site).

B-Sphericity: For ANOVA approaches, report Mauchly’s test and apply Greenhouse-Geisser correction when violated.

**Response:** Thank you for your suggestion. I added Table 5. Results of the sphericity (Mauchly) and handling of violations (Greenhouse-Geisser).

C- Correlation vs regression: Pearson r on aggregated mean values is misleading. Use subject-level paired BF–ISQ data and mixed regression to quantify association while adjusting for covariates. Report regression coefficient (β) interpreted as ISQ change per unit N BF, with 95% CI.

**Response:** Thank you for your comment. I was adding detailed reporting of how these analyses were conducted in the revision. I will rewrite the statistical analyses in Table 8, differences in regression coefficients (b) for BF and implant ISQ between the time, location of implants, and gender. b illustrated the mean percentage change in one unit of influencing variables (time, location of implants, and gender) based on the change in BF and implant ISQ. Significant differences in the regression coefficients (b) are denoted by * (p < 0.05). The term ratio reflects the quotient of bISQ / bBF.D

D- Multiple testing: Many comparisons presented (sex, site, time, post hoc) — control false discovery (e.g., Bonferroni or report adjusted p-values) or focus on prespecified primary comparison.

**Response:** Thank you for your question. I was writing a new analysis of these data, depending on intergroup comparisons of mean bite force and stability in both genders by using the Bonferroni test after adjustment of other factors in revised Table 6.

**Ethics, consent, data sharing— required clarifications**

A- Confirm ethics committee approval number and date; include a statement that the protocol was prospectively registered if applicable.

**Response:** Thank you for your valuable feedback. I added the ethics committee name, reference number, and date of approval as per journal requirements.

**This is revised**

All procedures performed in this study involving human participants were in accordance with the Declaration of Helsinki and its later amendments for human research. The study was conducted in accordance with and approved by the Ethics Committee of the College of Dentistry, University of Al-Ameed, Iraq (#52017, 20 February/2021). Written informed consent was obtained from all subjects and/or their legal guardian (s), where all adult participants entered the study after they received full information about the nature, aims, processes of the study, data sharing, the anonymization of participants in deposited data, and publication of raw data (without containing the name of participants) before signing an informed written Consent form.

B- Consent: clarify whether consent included data sharing and the form of anonymization for deposited data.

**Response:** Thank you for your valuable feedback. I was added to the consent, and all adult participants entered the study after they received full information about the nature, aims, processes of the study, data sharing, and the anonymization of participants in the deposited data.

**This is revised**

Written informed consent was obtained from all subjects and/or their legal guardian (s), where all adult participants entered the study after they received full information about the nature, aims, processes of the study, data sharing, the anonymization of participants in deposited data, and publication of raw data (without containing the name of participants) before signing an informed written Consent form.

C- Data availability: provide the exact repository link and DOI (supporting files are referenced, but specify where and how to access raw data and code to reproduce analyses).

**Response:** Thank you for your valuable feedback.

**This is revised**

**Supporting information**

S1 File. Data for analysis. doi: https://doi.org/10.6084/m9.figshare.29369663.v1

(XLSX) The data contains the recording of the bite force of the male and female participants after the insertion of dental implants for the anterior and posterior regions of the jaws. The dental implants' stability was monitored at the anterior and posterior regions of both genders' jaws. Data are available under the terms CC0.

**Response to the comment of reviewer 5**

**1. Response to comment: 1. Title and abstract**

- Abstract must mirror revised primary outcome and analytic approach (state adjusted analyses and main effect estimates with CIs). Avoid causal phrasing (“impact on long-term success”) unless supported by study duration.

**Response:** Thank you for your suggestion. I revised the abstract with the primary outcome and analytic approach.

**This is revised**

**ABSTRACT**

**Background:** Since dental implants (DI) are endosseous implants that are inserted into the bone and periodontium, which ultimately support the occlusal load, a patient's bite force (BF) may overburden the supporting DI, leading to bone loss and DI failure. The purposes of this study were to explore the effect of the BF on DIs in different locations of jaws, of both genders during three visits, to investigate DI stability in different locations of jaws, of both genders during three visits, and to assess the association of BF and DI stability with the effect of variables (time, implant location, and gender).

**Methods:** The current cohort study involved 80 individuals of both genders who had lost some teeth and needed DIs for the anterior and posterior regions of the jaws. After the insertion of the DIs, their BF was examined by the Loadstar™ sensor, and the DI stability was monitored at three visits of these individuals. First visit during the day of insertion of the crown on the abutment of the implant (immediately loading), second visit at six months following insertion, and third visit after 18 months following insertion.

**Results:** The data on the anterior and posterior bite forces (BFs) for the male and female participants after the insertion of the DIs were analysed at three visits. On average, the males exhibited a significantly higher posterior BF compared to the females at the respective three visits. Similarly, the average posterior ISQ in the males was significantly increased that in the females at the respective three visits. Results of the two-way ANOVA of all groups for the implant B and ISQ values were significantly influenced by the interaction of time, locations of the implants, and gender. The association between the BF and DI stability was significant in using the difference in the regression coefficient (𝑏), which was impacted by time, implant location, and gender.

**Conclusions:** This study found the significantly complex interaction of factors( time, location, gender) influencing on change of BF and ISQ, by affecting the process of ossteointegration. The significantly higher BF in the posterior regions of females and males, and significantly greater stability of DIs, could have a potential role in the best DIs therapy. In addition, the association of the BF and DI stability is significantly established with the most important factors influencing the change in BF and DI stability. It varies dynamically as the interface between the bone and the implant matures, and the patient's sex, time, and anatomical location all play a significant role in its context. The DIs that are loaded early are dependent on the BF. The BF may be crucial in determining DI stability.

**2. Response to comment: 2. Introduction**

- Tighten literature synthesis and explicitly identify the primary hypothesis and primary outcome measure.

**Response:** Thank you for pointing that out. I was rewriting the primary hypothesis and primary outcome measure.

**This is revised**

This study aims to explore the effect of the BF on DIs in different locations of jaws, of both genders, during three visits, to investigate DI stability in different locations of jaws, of both genders, during three visits, and to assess the association of BF and DI with the effect of variables(time, implant location, and gender).

The hypothesis is that there was a change in BF and ISQ of DIs during 18 months, followed insertion of the crown on the abutment of the implant. The null hypothesis is that there was no change in BF and ISQ of DIs during 18 months, following insertion of the crown on the abutment of the implant.

**3.Response to comment:3. Methods — participants**

- Clarify exact inclusion/exclusion items and present a CONSORT-style (STROBE-adapted) flow diagram.

- Provide rationale for age range and reasons for excluding smokers/systemic disease (possible selection bias).

**Response:** Thank you for your valuable feedback. I will revise the inclusion/exclusion precisely and remove contradictory phrasing to include a clear sentence and add a flow diagram. Adding patients aged 25-40 years, who had sufficient bone volume for DI placements, and excluding smokers/systemic because these represent confounding factors and affect the result. It is placed within the limitations of this study.

**4.Response to comment:4. Methods — implants and procedures**

- Give per-implant details: number of implants per patient, jaw (maxilla/mandible), tooth position, implant brand/size distribution, abutment type, immediate loading protocol specifics. State whether ISQ was measured before and after prosthetic insertion.

**Response：** Thank you for this suggestion. I added Table 1 summarizing implant characteristics (diameter, length, and number of implants in each size and group). Further surgical procedure (immediate loading protocol details.

**This is revised**

Table 1. Demographic data.

|  | DIs for the anterior regions of males | DIs for the posterior regions of males | DIs for the anterior regions of females | DIs for the posterior regions of females | p value |
| --- | --- | --- | --- | --- | --- |
| Number of patients (total) | 20 | 20 | 20 | 20 | 0.11 |
| Age | 35 | 37 | 36 | 34 | 0.18 |
| Gender | 20 | 20 | 20 | 20 | 0.21 |
| Number of implants (total) | 45 | 76 | 43 | 71 | 0.79 |
| Implant diameter |  |  |  |  |  |
| 3.3 mm | 8 | 10 | 7 | 12 | 0.122 |
| 3.5 mm | 19 | 22 | 23 | 21 | 0.084 |
| 3.8 mm | 14 | 24 | 11 | 30 | 0.056 |
| 4 mm | 4 | 20 | 2 | 8 | 0.077 |
| Length |  |  |  |  |  |
| 9 mm | 13 | 49 | 15 | 42 | 0.095 |
| 10 mm | 25 | 22 | 19 | 23 | 0.082 |
| 11 mm | 7 | 5 | 9 | 6 | 0.112 |

Surgical Procedure

The implants distributed were placed in the anterior and posterior regions of the jaws (Table 1). Following dental cone-beam computed tomography (CBCT), the quantity and quality of bone were assessed, and a stent was created for implantation at the proper location for each patient. No bone augmentation treatment was used throughout the implant placement process. As instructed by the manufacturer, the location was drilled using a point Lindemann drill first, then surgical drills. A skilled researcher meticulously drilled every dental implant bed at a consistent length and angles in order to produce comparable insertion torque values (ITV) of about 35 Ncm amongst the implants. A drilling machine specifically made for implant surgery was used for measuring ITV as much as 35 Ncm at around 20 rpm and 8 Hz. The drill unit's handpiece was the only tool used to put each implant. Following surgery, CBCT was utilized to assess the integrity of the osseous tissue surrounding the implant [28].

**5.Response to comment:5. Methods — BF measurement**

- Describe Loadstar calibration, sensor placement protocol (how measured for anterior vs posterior), number of repeated bites averaged, and units (N). Report measurement reliability (intra-operator / test-retest) if available.

**Response:** Thank you for your comment. I was adding detailed reporting of how the Loadstar calibration, sensor placement protocol, number of repeated bites averaged, and units were conducted in the revision. The reliability of measurements was estimated by intra-operator evaluation and repeated test with the method of maximum bite force estimation, which was performed three times on both sides, separated by two minutes, and the average result for every side.

**This is revised**

Bite Force (BF) Assessment

Bite force (BF) assessments were acquired utilising a Loadstar™ sensor (Loadstar sensor, DI-100U,16-bit load cell interface, Fremont, California) (Figure 1), which offers various force measurement devices with updated data rates of up to 50 KHz. This capability might be advantageous for applications such as failure-strength testing or material characterisation. The BF values were tested within the range of 100-400 N at particular time intervals. This systematic method was observed to guarantee standardization and dependability in the assessment of the BF. The protocol of maximum bite force estimation. The individual was told to sit on a dental seat without a head support, erect, comfortable, and unstrained. A Loadstar sensor was used for determining the maximum force of biting (MBF) and vertically inter-occlusal BF on both sides. A USB cable is used to link the sensor to the PC. The first molars region (right and left) was where the load sensor was placed horizontally. Participants were instructed to bite as heavily as they could onto the load sensor for a brief period of time. A new record—the greatest—was established every second. Ten measurements of the recording table were selected. This method was performed three times on both sides, separated by two minutes, and the average result for every side was obtained as MBF. We calculated the maximal BF in Newtons [29]. The reliability of measurements was estimated by intra-operator evaluation and repeated test with the method of MBF.

**6.Response to comment:6. Methods — stability measurement**

- Describe ISQ measurement protocol (Smartpeg type, measurement directions, operator blinding) and handling of multiple ISQ readings per implant.

**Response:** Thank you for your question. I revised stability measurements and added the ISQ measurement protocol.

**This is revised**

Stability measurements

The method for monitoring implant stability during healing and immediately loading is based on resonance frequency analysis (RFA) applied to the implant–bone interface. Devices using this method contain a transducer peg, which is connected to the implant and excited by magnetic waves over a range of frequencies. The frequency of the resultant vibration is automatically translated into an index called the implant stability quotient (ISQ), with values ranging between 0 and 100. The RFA values are a measure of the deflection of the implant–bone complex by the lateral forces applied by the transducer and reflect the multidirectional fixation strength. The stability of each implant (one measurement from each of the 3 different directions) was measured with the Osstell (Integration Diagnostics AB, Göteborg, Sweden). The Osstell system is a magnetic detection device. RFA devices after the transducer (Smartpeg) was screwed to the implant to obtain the ISQ.

The protocol of measurement of DI stability, an experienced, right-handed investigator, assessed the ISQ. ISQ was assessed using the Osstell in order to keep the fixing force of the implant from altering when the healing abutment was being installed and released. The smart peg was manually attached to the implant fixture in order to use the Osstell ISQ Mentor for evaluation. Every equipment was operated in compliance with the guidelines provided by the manufacturer. The Osstell ISQ mentor's manufacturer advises holding the gadget tip at a 45-degree angle and close (2.0–4.0 mm) to the smart peg surface, avoiding contacting it. The implants in each location were evaluated one after the other since testing a single implant twice could increase the precision of measurements. After the measurements with the Osstell ISQ Mentor, healing abutments were connected to the implant with 30 Ncm torque, using a torque ratchet, by selecting a height that could expose about 2.0 mm from the gingival level [30].

**7. Response to comment: 7. Statistical analysis**

- Replace current description with explicit mixed-effects model plan: formula, fixed and random effects, covariates, model selection, diagnostics, and multiple testing control. Provide software and package versions used. Report how missing data were handled (e.g., mixed models assume MAR; list any imputation).

**Response:** Thank you for your valuable feedback. I will revise the data tables to include a clear sentence in green and add the tables4,5 for further clarification. Table 4. Results of the two-way ANOVA of all groups for the implant bite forces and stability values between the time, locations of the implants, and gender with mixed-effects models. Adding the interaction terms (time × sex, time × site, BF × site). If any patient in this study missed a follow-up visit, the author would call the patient to reschedule a new appointment.

**This is revised**

Statistical Analyses

Descriptive statistics through GraphPad® Prism 9.5.1 were used to summarize the patient demographics, BF measurements, and DI stability measurements. The normality of distribution (parametric)was significantly result by used the Shapiro–Wilk. The parametric data was analyzed. Then the continuous variables were expressed as the mean standard deviation (±), while the categorical variables were presented as frequencies and percentages. Comparative analyses, such as an analysis of variance (One Way Repeated-Measures ANOVA), were performed to assess differences in the BF and DI stability in males and females at the anterior and posterior areas of the jaws among three postoperative time points. The two-way and three-way ANOVA of all groups for the implant bite forces and stability values between the time, locations of the implants, and gender. The sphericity (Mauchly) and handling of violations (Greenhouse-Geisser)were employed. For comparisons between more than two groups, the Bonferroni test for bite force and stability was used. A Pearson correlation was done to investigate the relationship between the BF and DI stability, and *P* < 0.05 was considered statistically significant. The mixed-effects regression to model ISQ ~ BF + time + location + gender and fixed effects, 95% CIs. If any patient in this study missed a follow-up visit, the author would call the patient to reschedule a new appointment.

**8.Response to comment:**8. Results — descriptive

- Provide baseline table with patient characteristics by sex and by implant site; include implant-level counts and per-timepoint sample sizes.

- Clean up Tables 1–3: ensure all values are mean ± SD with units, n per cell, and clear p-values and test statistics.

**Response：** Thank you for this suggestion. I added a table 1 summarizing descriptive data of patient demographics (number of patients, age, gender, number of implants, implant diameter, and length. The age and gender of participants and implant number, diameter, and length were non significantly different between study groups. Adding, I cleaned up Tables 1–3, including the mean ± SD with units, n per cell, and clear p-values and test statistics.

**This is revised**

Results

The current study enrolled 128 individuals, and the inclusion criteria were present in 80individuals needing DIs for the anterior and posterior regions of the jaws in both genders (Figure 2). Descriptive data of patient demographics (number of patients, age, gender, number of implants, implant diameter, and length. The age and gender of participants and implant number, diameter, and length were non significantly different between study groups (Table 1)

**9.Response to comment:**9. Results — main analyses

- Present adjusted mixed-model estimates (effect of BF on ISQ) with β, 95% CI, p-value. Provide interaction tests (BF × sex, BF × site). If the interaction is non-significant, present pooled estimates and exploratory stratified analyses.

**Response:** Thank you for your valuable feedback. I will revise the data tables to include a clear sentence in green and add the tables4,5 for further clarification. Table 4. Results of the two-way ANOVA of all groups for the implant bite forces and stability values between the time, locations of the implants, and gender with mixed-effects models. Adding the interaction terms (time × sex, time × site, BF × site). Table 8 Differences in regression coefficients (b) for BF and implant ISQ between the time, location of implants, and gender. b illustrated the mean percentage change in one unit of influencing variables (Time, Location of Implants, and Gender) based on the change in BF and implant ISQ. Significant differences in the regression coefficients (b) are denoted by * (p < 0.05). The term ratio reflects the quotient of bISQ / bBF.

**10.Response to comment:**10. Results — figures

- Provide subject-level scatterplots (BF vs ISQ) with model-predicted lines and 95% CI; include residual diagnostics in supplement.

**Response:** Thank you for your valuable feedback. I added figure 2. The CONSORT 2010 flow diagram, and clear other figures.

**11. Response to comment:** 11. Discussion

- Restructure: start with main findings (with adjusted estimates), compare to prior studies, discuss biological plausibility, limitations (selection bias, short follow-up, residual confounding, measurement error), and implications.

- Remove causal wording; state that BF is associated with ISQ changes and that further, longer-term and interventional studies are needed.

**Response:** Thank you for your valuable feedback. I will revise the discussion with executed analyses and limitations to include a clear sentence for further clarification.

**12. Response to comment:**12. Limitations

- Expand: explain possible selection bias from excluding smokers/systemic disease, limited follow-up for “long-term” claims, and lack of randomization.

**Response:** Thank you for your comment. We were excluding smokers/systemic disease from the study because these represent confounding factors and affect bone metabolism. The participants should have sufficient bone volume and density for DI placements. It is placed within the limitations of this study.

**13. Response to comment:**13. Conclusion

- Rephrase to reflect association rather than causation; avoid overreaching on “long-term success”.

**Response:** Thank you for your valuable feedback. I revised the conclusion.

**Conclusion**

This study found a significantly complex interaction of influencing factors, time, location, and gender on the change of BF and ISQ by affecting the process of ossteointegration. The significant changes of time, location, and gender during 18 months influence an increase in BF, and changeISQ with (primary, secondary) stability. The significantly high mean BF in the posterior regions of males and females after delivery of dental implant restoration, and the significantly higher mean stability of DIs during the 18 months following insertion of the crown, within three follow-up visits, could play a potential role in great DIs therapy. This study found a significant association between BF and DI stability with the impact of time, location of implants, and gender. The BF was higher on the posterior DIs area in both genders due to different occlusion circumstances primarily affect the amplitude and direction of the BF. Besides, the result of DI stability revealed that it increased in the posterior regions of females and males compared to the anterior regions due to denser bone quality. It clarified that the effective incorporation of the DI into the bone was important for the establishment of the primary and secondary stability.

In addition, the relationship between the BF and DI stability is significantly established, with the most important factors influencing the change in BF and DI stability. It varies in a dynamic manner as the interface between the bone and the implant matures, and the patient's sex, time, and anatomical location all play a significant role in its context. Moreover, the understanding of the BF impact and ensuring the stability can help implement appropriate treatment strategies and the best therapy for DIs.

**Special thanks to you for your good comments.**

We tried our best to improve the correction of the manuscript and made some changes in the revision manuscript. These changes will not influence the content and framework of the paper. And here we highlight the changes marked in green in the revised paper.

We appreciate for Editors and Reviewers’ warm work earnestly, and hope that the correction will meet with approval.

Once again, thank you very much for your comments and suggestions.

We look forward to hearing from you. If you have any queries, please don’t hesitate to contact me anytime at the address below.

Thank you and best regards.

Yours sincerely,

14. Supplementary material

- Provide a cleaned dataset and analysis script (R, SAS, SPSS) or at least provide sufficient summary statistics to reproduce results.

**Supporting information**

S1 File. Data for analysis.

doi: https://doi.org/10.6084/m9.figshare.29369663.v1

(XLSX), The data contains the recording of the bite force of the male and female participants after the insertion of dental implants for the anterior and posterior regions of the jaws. The dental implants' stability was monitored at the anterior and posterior regions of both genders' jaws. Data are available under the terms CC0.
